# Uncertainties in Interpolated Spectral Data

**DOI:** 10.6028/jres.108.007

**Published:** 2003-02-01

**Authors:** James L. Gardner

**Affiliations:** CSIRO National Measurement Laboratory, Lindfield, Australia 2070

**Keywords:** interpolation, photometry, radiometry, uncertainty

## Abstract

Interpolation is often used to improve the accuracy of integrals over spectral data convolved with various response functions or power distributions. Formulae are developed for propagation of uncertainties through the interpolation process, specifically for Lagrangian interpolation increasing a regular data set by factors of 5 and 2, and for cubic-spline interpolation. The interpolated data are correlated; these correlations must be considered when combining the interpolated values, as in integration. Examples are given using a common spectral integral in photometry. Correlation coefficients are developed for Lagrangian interpolation where the input data are uncorrelated. It is demonstrated that in practical cases, uncertainties for the integral formed using interpolated data can be reliably estimated using the original data.

## 1. Introduction

Measurements of spectral irradiance, spectral responsivity or spectral reflectance are often made for a limited set of wavelengths and then used to calculate weighted spectral sums for photometry, colorimetry or filter radiometry. It is often necessary to interpolate the spectral data to a finer grid to avoid errors arising from the discrete approximation used to estimate the integral where the weighting function varies strongly between the wavelengths at which the measurements were made [1]. Interpolated values are then correlated to the original data and to nearby interpolated points; unless these correlations are taken into account, uncertainty calculations will give misleading results, generally underestimating errors in the spectral sums by significant amounts.

Interpolation may also be required when reference standards are provided for a limited set of wavelengths. Many of the primary reference standards in the national metrology institutes are derived in a functional form, and can be calculated on as fine a wavelength grid as required, along with all the necessary correlations [2]. However, calibrations of client lamps or detectors involve a transfer, by comparison, to artifacts that do not necessarily show a spectral variation that is easily modeled, and in the interests of reducing costs may be provided on a limited number of wavelengths. The reference data at different wavelengths may also be correlated. While the primary reference standards are often strongly correlated between wavelengths, the transfer process itself adds uncertainty that is generally random and often reduces the correlations to negligible levels [2]

Where data are available at sufficient wavelengths to avoid errors due to the sum approximation to the integral, it is preferable to interpolate reference tables, such as the photometric response function *V*_λ_ and the colorimetric tristumulus response functions [3] to the wavelengths at which measurements are taken, because those tables contain no uncertainty. However, interpolation of measurement data is often applied. One reason is that software for a calculation may require data at a particular interval, another is that instrument measurement programs may provide limited sets of data.

Following a brief description of uncertainty propagation, this paper is divided into two sections. The first covers Lagrangian interpolation, the second cubic spline interpolation; these are the two most commonly used interpolation methods. In each section, interpolation from a data set which is itself correlated is considered. Various simplifications for practical applications are made, and examples are presented. A conclusion is that in practical terms, uncertainty can be accurately derived from the original data set without a complex calculation of correlations.

## 2. Propagation of Uncertainty

Uncertainty propagation is described in detail in the ISO *Guide to the Expression of Uncertainty in Measurement* [4]. The uncertainty in a quantity *y* formed by combining measured quantities *x*_i_ through the relationship y = *f* (*x*_1_, *x*_2_,..*x_N_*) is given by

$$u^{2}\left(y\right)=\sum_{i=1}^{N}{\left(\frac{\partial f}{\partial x_{i}}\right)}^{2}u^{2}\left(x_{i}\right)+\sum_{i=1}^{N}\sum_{j\neq i=1}^{N}\frac{\partial f}{\partial x_{i}}\frac{\partial f}{\partial x_{j}}u\left(x_{i},x_{j}\right),$$ (1)

where *u*(*x_i_*) is the uncertainty in *x_i_* and *u*(*x_i_*, *x_j_*) is the covariance between *x_i_* and *x_j_*. For uncorrelated input quantities, the covariance between pairs of variables is zero and Eq. (1) reduces to the "sum of squares" commonly applied. The derivatives ∂*f* / ∂*x_i_* are sensitivity coefficients for the dependence of *y* on the various measured quantities. Given that *u*^2^ (*x_i_*) ≡ *u*(*x_i_*, *x_i_*), Eq. (1) can be expressed as

$$u^{2}\left(y\right)=f_{y}^{T}U_{x}f_{y},$$ (2)

where

$$f={\left(\frac{\partial f}{\partial x_{1}}\frac{\partial f}{\partial x_{2}}..\frac{\partial f}{\partial x_{n}}\right)}^{T}$$ (3)

is a column vector of sensitivity coefficients (^T^ indicates the transpose) and

$$U_{x}=\left[u\left(x_{i},x_{j}\right)\right]$$ (4)

is the *N*× *N* uncertainty matrix.

Interpolation of spectral data is generally performed to produce a set of values (*p_k_*, *x_k_*) from set (*y_i_*, *x_i_*) where *x_i_* is the independent variable (generally wavelength in radiometry). Quantities in the set (*p_k_*, *x_k_*) that depend on the same (*y_i_*, *x_i_)* are correlated through this common dependence. The covariance between two values *p_k_* and *p_m_* (and, when *k* = *m*, the square of the uncertainty) is given by

$$u\left(p_{k},p_{m}\right)=\sum_{i=1}^{N}\sum_{j=1}^{N}\frac{\partial p_{k}}{\partial y_{i}}\frac{\partial p_{m}}{\partial y_{j}}u\left(y_{i},y_{j}\right).$$ (5)

In matrix form, this is simply expressed as

$$u\left(p_{k},p_{m}\right)=f_{p_{k}}{\;}^{T}U_{y}f_{p_{m}}.$$ (6)

It is sometimes convenient to use correlation coefficients rather than covariances, defined as

$$r\left(p_{k},p_{m}\right)=\frac{u\left(p_{k},p_{m}\right)}{\sqrt{u\left(p_{k},p_{k}\right)u\left(p_{m},p_{m}\right)}}.$$ (7)

A matrix of correlation coefficients is square, symmetric about the diagonal and has value 1 in the diagonal elements.

## 3. Lagrangian Interpolation

We have a tabulated function *y_i_* at values *x_i_*:

$$y_{i}=y\left(x_{i}\right),i=1\ldots .N.$$ (8)

For *x* in the range *x_m_* to *x_m_*_+_*_n_*, the formula for Lagrangian interpolation is [5]

$$\begin{array}{l} p\left(x\right)=\sum_{j=1}^{n}\frac{\prod_{i\neq j=1}^{n}\left(x-x_{m+i-1}\right)}{\prod_{i\neq j=1}^{n}\left(x_{m+j-1}-x_{m+i-1}\right)}y_{m+j-1}\\ \;=\sum_{j=1}^{n}w_{j}y_{m+j-1} \end{array}.$$ (9)

Equation (9) represents an (*n*−1)th order polynomial fitted through the *n* original values. Interpolated data are formed as a linear combination of nearby existing data. Sensitivity coefficients for the dependence of the interpolated data on the input data are simply the weights *w_j_* in Eq. (9). Covariances between the output values, that is, the original input values plus the interpolated values, take several forms. Correlations present in the original values remain. The values newly formed by interpolation are correlated to both the input values forming them (and through them to the remaining input values if correlations are present in the original set) and to any new values formed from common input values. All of these covariances, including the uncertainty of the interpolated values, can be calculated through Eq. (6).

In many instances, the input data *y_i_* are present at regular values of *x_i_* and further, the output data are also required at regular intervals. Interpolation is usually then performed by running Eq. (9) through the data and forming a new value or values in the center of the range. The multipliers convolving the data are determined by the interval spacings only and can be calculated prior to the interpolation. This was clearly demonstrated by Savitsky and Golay [6] in the development of their algorithms for smoothing and differentiation of spectral data where polynomial expressions of various order are fitted to regularly-spaced data; in those cases, the coefficients are determined as fixed linear combinations of the input dependent data. The arguments presented here for Lagrangian interpolation can easily be extended to cover smoothing of data using the Savitsky-Golay routines. Two-point Lagrangian interpolation forming a new value in the center of the existing values has weights (*w*_1_, *w*_2_)=(½, ½), equivalent to a linear interpolation.

**Fig. 1:**
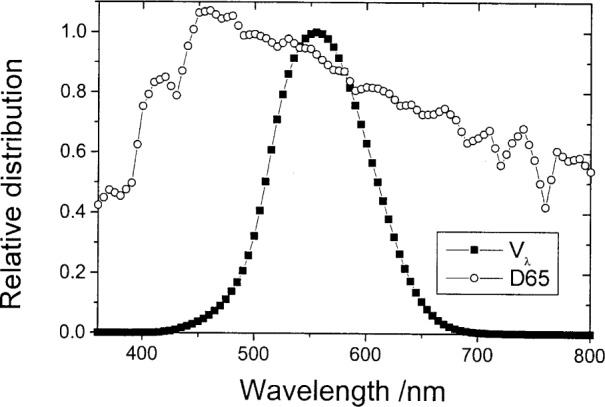
Distributions CIE V*_λ_* and D65 used for interpolation examples.

Propagation of uncertainty through two common examples of Lagrangian interpolation used in photometry are now discussed. In both of these we consider the calculation of illuminance response to CIE illuminant D65, a tabulated distribution carrying no uncertainty, for a photometer whose spectral response is a close approximation to V*_λ_*, measured at different spectral intervals and where the measured response values are uncorrelated. These distributions are shown in Fig. 1 for a wavelength interval of 5 nm. As the response function tapers to zero at each end, the luminance response is given as

$$R_{V}=\Delta \lambda \sum_{i=1}^{N}R_{i}E_{D65,i},$$ (10)

where *R_i_* is the photometer response and *E*_D65,_*_i_* is the illuminant value at the *i*th wavelength, respectively, and Δ*λ* is the 5 nm wavelength separation between the values.

For uncorrelated spectral response values, the uncertainty in *R_v_* is given by

$$u^{2}\left(R_{V}\right)={\left(\Delta \lambda \right)}^{2}\sum_{i=1}^{N}E_{D65,i}^{2}u^{2}\left(R_{i}\right).$$ (11)

For values as tabulated by CIE [3], the value of *R_v_* is 10567.41, with an uncertainty 19.60 (relative uncertainty 0.1855 %) if the responsivity values have a relative uncertainty of 1 % and are uncorrelated. (Note that extra significant figures for the uncertainty are presented above those of normal practice for the purpose of comparison.)

### 3.1 Photometer Measured at 10 nm Intervals, Interpolated to 5 nm

The spectral integral Eq. (10) for input values on a 10 nm grid evaluates to 10568.18, a small change compared to the 5 nm data due to the discrete approximation to the integral; the relative uncertainty for uncorrelated spectral response values with a relative uncertainty of 1 % becomes 0.2623 %, or the expected $\sqrt{2}$ increase compared to the response measured at 5 nm intervals. We wish to interpolate the photometer response with a four point Lagrangian function to data on a 5 nm interval. The weights for Eq. (9) are then

$$w_{1},w_{2},w_{3},w_{4}=\frac{-1}{16},\frac{9}{16},\frac{9}{16},\frac{-1}{16}$$ (12)

and, as the input data are uncorrelated, the uncertainty for an interpolated value is given by

$$u^{2}\left(p\right)=\sum_{j=1}^{4}w_{j}^{2}u^{2}\left(y_{m+j-1}\right).$$ (13)

In any given interval spanning four input values, the uncertainty value *u* (*y_i_*) is approximately constant, and the interpolated values have an uncertainty approx. 80 % of the original values in that range. If we ignore the correlations that have been introduced, the relative uncertainty of the integral evaluates as 0.17 %, low compared to the original value and clearly incorrect as it would imply that it could be reduced to zero by repeated interpolation.

A full correlation matrix for the output values is formed as follows. First the interpolation is performed (using linear interpolation in the first and last intervals of the input values). From *N* original values, we now have (2*N*−1) values

$$\begin{array}{l} y_{i}=y_{\text{original},j};i\text{odd},j=\left(i+1\right)/2\\ y_{i}=w_{1}y_{i-3}+w_{2}y_{i-1}+w_{3}y_{i+1}w_{4}y_{i+3}\\ 4<i<2N-4,\;i\;\text{even}. \end{array};$$ (14)

Uncertainties for these values are known for the original data and for the interpolated data from Eq. (13) and these are used to populate the diagonal elements of the uncertainty matrix ***U****_y_*. We then have to populate the elements to the right of the diagonal only before filling to the left of the diagonal by symmetry. As the original input values (now at *i* odd) are uncorrelated, we have for all *i*, from Eq. (5),

$$\begin{array}{c} u\left(y_{i},y_{i+1}\right)=w_{3}u^{2}\left(y_{i+1}\right)\\ u\left(y_{i},y_{i+3}\right)=w_{4}u^{2}\left(y_{i+3}\right) \end{array}$$ (15)

For *i* even (interpolated values),

$$\begin{array}{c} y_{i+2}=w_{1}y_{i-1}+w_{2}y_{i+1}+w_{3}y_{i+3}+w_{4}y_{i+5}\\ y_{i+4}=w_{1}y_{i+1}+w_{2}y_{i+3}+w_{3}y_{i+5}+w_{4}y_{i+7}\\ y_{i+6}=w_{1}y_{i+3}+w_{2}y_{i+5}+w_{3}y_{i+7}+w_{4}y_{i+9} \end{array},$$ (16)

and from Eq. (5) we have

$$\begin{array}{l} u\left(y_{i},y_{i+2}\right)=w_{1}w_{2}u^{2}\left(y_{i-1}\right)+w_{2}w_{3}u^{2}\left(y_{i+1}\right)+w_{3}w_{4}u^{2}\left(y_{i+3}\right)\\ u\left(y_{i},y_{i+4}\right)=w_{1}w_{3}u^{2}\left(y_{i+1}\right)+w_{2}w_{4}u^{2}\left(y_{i+3}\right)\\ u\left(y_{i},y_{i+6}\right)=w_{1}w_{4}u^{2}\left(y_{i+3}\right) \end{array}.$$ (17)

For completeness, similar expressions were applied for the values formed by linear interpolation in the first and last intervals. Uncertainty calculated for the integral including all these correlations was then exactly that calculated with the original data for the 10 nm grid.

One further simplification can be made for uncorrelated input values. In practical terms, nearby values have the same uncertainty. Hence the sets of Eqs. (14) to (17) can be reduced to a matrix of correlation coefficients determined purely from the weights,

(..      1916463164−1164−1816401164 191640−116400  1916463164−1164−18164   191640−1164    ..  ), (18)

where the first row corresponds to an interpolated value. Uncertainty for the integral using the interpolated data set is then found by modifying the sensitivity column vector to include the uncertainty at each value,

$$f=\left(\frac{\partial f}{\partial y_{i}}u\left(y_{i}\right)\right),$$ (19)

and then performing the matrix multiplication Eq. (2). A negligible change relative to the true values is due to averaging through regions where the response is changing rapidly; while the relative uncertainty in these regions is constant, the absolute value is not.

Table 1 shows uncertainties calculated for all the options discussed in this section. It can be seen that proper accounting for the correlations introduced by the interpolation reproduces the uncertainty calculated for the input values alone, and that using the correlation coefficients provides a practical calculation of the uncertainty matrix.

### 3.2 Photometer Measured at 5 nm Intervals, Interpolated to 1 nm

After re-arranging the *N* input values to their new positions, we add four values between input values *y_i_* and *y_i_*_+5_ that can be represented as

$$\left(\begin{array}{l} y_{i+1}\\ y_{i+2}\\ y_{i+3}\\ y_{i+4} \end{array}\right)=\frac{1}{125}\left(\begin{array}{rrrr} -6 & 108 & 27 & -4\\ -8 & 84 & 56 & -7\\ -7 & 56 & 84 & -8\\ -4 & 27 & 108 & -6 \end{array}\right)\left(\begin{array}{l} y_{i-5}\\ y_{i}\\ y_{i+5}\\ y_{i+10} \end{array}\right).$$ (20)

**Fig. 2:**
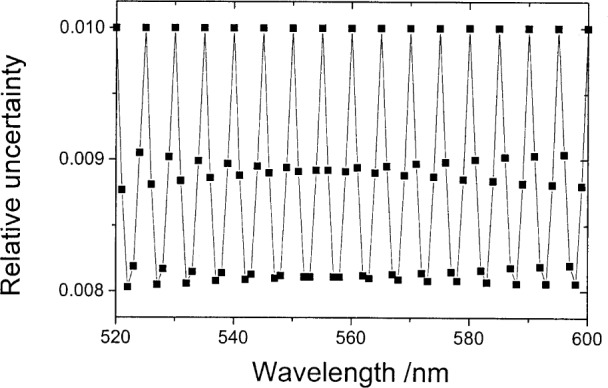
Relative uncertainties for the interpolated V*_λ_* data set. Original values assumed uncorrelated, with a relative uncertainty of 1 %.

Fig. 2 shows the relative uncertainty of the central data set where a photometer with a V*_λ_* response is interpolated from 5 nm to 1 nm and the input values are assumed uncorrelated with a relative uncertainty of 1 %. Uncertainties of the interpolated values are lower than those of the input values. If we ignore the correlations introduced by the interpolation, the relative uncertainty of the integral with the D65 illuminant (interpolated to a 1 nm grid with a cubic-spine routine), reduces to 0.074 %, a value too low by the order of $\sqrt{5}$. The integral itself has the same value as that shown in Table 1 for the 5 nm grid.

For a four point Lagrange interpolation adding four values between each of the input values, correlations in the output set extend over the 19 values following an input value. The matrix of correlation coefficients is shown as the transpose relative to Eq. (18) in the interests of printing; that is, it is equivalent to filling to the lower left of the diagonal of the correlation matrix prior to filling the upper right by symmetry. Beginning at a column corresponding to an input value, the matrix of correlation coefficients is

(..       1      1085⋅24891     845⋅206121322489⋅20611    565⋅206116782489⋅206116782061⋅24891   275⋅24891176248916782061⋅248921322061⋅24891  0275⋅2489565⋅2061845⋅20611085⋅2489.. 275⋅248931824898612061⋅248914402061⋅248920162489  565⋅20611322489⋅20615322061976206114402489⋅2061  845⋅206172489⋅2061245206153220618612489⋅2061  1085⋅2489−67248972061⋅248913224893182489  0−45⋅2489−75⋅2061−85⋅2061−65⋅2489  0−1142489−2102061⋅2489−2642061⋅2489−2522489  0−1042489⋅2061−1962061−2562061−2642489⋅2061  0−772489⋅2061−1472061−1962061−2102489⋅2061  0−402489−772061⋅2489−1042061⋅2489−1442489  00000  0245⋅2489425⋅2061⋅2489485⋅2061⋅2489365⋅2489  0325⋅2489⋅2061565⋅2061645⋅2061485⋅2489⋅2061  0285⋅2489⋅2061495⋅2061565⋅2061425⋅2489⋅2061  0165⋅2489285⋅2061⋅2489325⋅2061⋅2489245⋅2489  00000 ). (21)

A number of these, for the values furthest from the input set, are negligible. The relative uncertainty of the integral for the interpolated data set calculated with these correlation coefficients is 0.197 %, equivalent in practical terms to that calculated using the original 5 nm data set and shown in Table 1.

## 4. Cubic-Spline Uncertainty Propagation

For our set of data Eq. (8), cubic-spline interpolation [7] calculates a value *y* at *x* in the interval *x_i_* to *x_i_*_+1_ as

$$y=Ay_{i}+By_{i+1}+Cy_{i}^{\prime \prime }+Dy_{i+1}^{\prime \prime },$$ (22)

where

$$A=\frac{x_{i+1}-x}{x_{i+1}-x_{i}},$$ (23)

$$B=\frac{x-x_{i}}{x_{i+1}-x_{i}},$$ (24)

$$C=\frac{1}{6}\left(A^{3}-A\right){\left(x_{i+1}-x_{i}\right)}^{2},$$ (25)

$$D=\frac{1}{6}\left(B^{3}-B\right){\left(x_{i+1}-x_{i}\right)}^{2}.$$ (26)

The first two terms of Eq. (22) represent simple linear interpolation. Including the second derivatives *y"* yields a function that has first and second derivatives continuous at the boundaries between intervals.

The second-derivatives are unknown. The relation between them is given by

$$\frac{x_{i}-x_{i-1}}{6}y_{i-1}^{\prime \prime }+\frac{x_{i+1}-x_{i-1}}{3}y_{i}^{\prime \prime }+\frac{x_{i+1}-x_{i}}{6}y_{i+1}^{\prime \prime }=\frac{y_{i+1}-y_{i}}{x_{i+1}-x_{i}}-\frac{y_{i}-y_{i-1}}{x_{i}-x_{i-1}},$$ (27)

which is a system of *N*−2 equations in the *N* unknowns $y_{i}^{\prime \prime }$. The natural cubic-spline, which is commonly used, sets

$$y_{i}^{\prime \prime }=y_{N}^{\prime \prime }=0$$ (28)

and solves for the remaining terms [7]. We are interested in using the cubic-spline interpolation on spectral data of known uncertainties, including the possibility of correlations, where the interpolated data may then be combined in various ways, so that not only the uncertainties in the interpolated data but also the correlations present are important in propagating uncertainties in the combinations.

The (*N*−2) values of $y_{i}^{\prime \prime }$ depend on each of the input values, i.e., are correlated to each input value. Then even for uncorrelated input data, the output data are correlated over the whole set of interpolated values. We wish to calculate the covariance *u* (*y_n_*, *y_m_*) between two interpolated values

$$\begin{array}{l} y_{m}=A_{m}y_{i}+B_{m}y_{i+1}+C_{m}y_{i}^{\prime \prime }+D_{m}y_{i+1}^{\prime \prime }\\ y_{n}=A_{n}y_{j}+B_{n}y_{j+1}+C_{n}y_{j}^{\prime \prime }+D_{n}y_{j+1}^{\prime \prime } \end{array},$$ (29)

where the *y_n_* and *y_m_* values may be in the same or different intervals denoted by *i*, *j*. The uncertainty in *y_n_* is given by

$$u^{2}\left(y_{n}\right)=u\left(y_{n},y_{n}\right).$$ (30)

The covariance between (and uncertainty of) the input values is known, carried in the matrix

$$U_{y}=\left[u\left(y_{i},y_{j}\right)\right].$$ (31)

To propagate uncertainties through Eq. (29) we need the covariance between the second-derivative values and the input values, and between the second-derivatives themselves. These in turn require the sensitivity coefficients for the dependence of the second-derivative on the input values, $\partial y_{k}^{\prime \prime }/\partial y_{i}$.

The set of *N*−2 equations Eq. (27) can be written in matrix form

$$\left(\begin{array}{c} \frac{y_{3}-y_{2}}{x_{3}-x_{2}}-\frac{y_{2}-y_{1}}{x_{2}-x_{1}}\\ ..\\ \frac{y_{i+2}-y_{i+1}}{x_{i+2}-x_{i+1}}-\frac{y_{i+1}-y_{i}}{x_{i+1}-x_{i}}\\ ..\\ \frac{y_{N}-y_{N-1}}{x_{N}-x_{N-1}}-\frac{y_{N-1}-y_{N-2}}{x_{N-1}-x_{N-2}} \end{array}\right)=\left(h_{i,j}\right)\left(\begin{array}{c} y_{2}^{\prime \prime }\\ ..\\ y_{N-1}^{\prime \prime } \end{array}\right),$$ (32)

where

$$\begin{array}{l} h_{i,i-1}=\frac{x_{i}-x_{i-1}}{6}\\ h_{i,j}=\frac{x_{i+2}-x_{i}}{3}\\ h_{i,i+1}=\frac{x_{i+2}-x_{i+1}}{6} \end{array}$$ (33)

is a tri-diagonal matrix with remaining terms zero. Multiplying both sides of Eq. (32) on the left by the inverse matrix ${\left(h_{i,j}\right)}^{-1}$ yields the required second-derivatives. By selecting the *i*th row of this multiplication we have the relationship

$$y_{i+1}^{\prime \prime }=\sum_{j=1}^{N-2}h_{i,j}^{-1}\left(\frac{y_{j+2}-y_{j+1}}{x_{j+2}-x_{j+1}}-\frac{y_{j+1}-y_{j}}{x_{j+1}-x_{j}}\right),$$ (34)

which when rearranged to

$$\begin{array}{l} y_{i+1}^{\prime \prime }=\sum_{j=3}^{N}h_{i,j-2}^{-1}\frac{y_{j+2}}{x_{j+2}-x_{j+1}}\\ \;-\sum_{j=2}^{N-1}h_{i,j-1}^{-1}\left(\frac{1}{x_{j+2}-x_{j+1}}\right)+\left(\frac{1}{x_{j+1}-x_{j}}\right)y_{j}\\ \;+\sum_{j=1}^{N-2}h_{i,j}^{-1}\frac{y_{j}}{x_{j+1}-x_{j}} \end{array}$$ (35)

yields

$$\begin{array}{l} \frac{\partial y_{i+1}^{\prime \prime }}{\partial y_{j}}=\frac{h_{i,j-2}^{-1}}{x_{j}-x_{j-1}}\\ \;-h_{i,j-1}^{-1}\left(\frac{1}{x_{j+1}-x_{j}}+\frac{1}{x_{j}-x_{j-1}}\right)\\ \;+\frac{h_{i,j}^{-1}}{x_{j+1}-x_{j}}. \end{array}$$ (36)

These are augmented by

$$\partial y_{k}^{\prime \prime }/\partial y_{i}=0,k=1,N.$$ (37)

If the input *y_i_* values are uncorrelated, the required covariances are given by

$$u\left(y_{i}^{\prime \prime },y_{j}^{\prime \prime }\right)=\sum_{k=1}^{N}\frac{\partial y_{i}^{\prime \prime }}{\partial y_{k}}\frac{\partial y_{j}^{\prime \prime }}{\partial y_{k}}u^{2}\left(y_{k}\right)$$ (38)

and

$$\begin{array}{l} u\left(y_{i}^{\prime \prime },y_{j}\right)=\sum_{k=1}^{N}\frac{\partial y_{i}^{\prime \prime }}{\partial y_{k}}\frac{\partial y_{j}}{\partial y_{k}}u^{2}\left(y_{k}\right)\\ \;=\frac{\partial y_{i}^{\prime \prime }}{\partial y_{k}}u^{2}\left(y_{k}\right) \end{array}$$ (39)

Note that the *N* × *N* matrix represented by Eq. (39) is not symmetric. For correlated inputs, the sums required are the matrix products

$$\begin{array}{l} u\left(y_{i}^{\prime \prime },y_{j}^{\prime \prime }\right)=f_{i}^{T}U_{y}f_{j}\\ u\left(y_{i}^{\prime \prime },y_{j}\right)=f_{i}^{T}U_{y}g_{j} \end{array},$$ (40)

where

$$f_{i}=\left(\frac{\partial y_{i}^{\prime \prime }}{\partial y_{k}}\right),k=1..N$$ (41)

is a column-vector of sensitivity coefficients of the second-derivatives vs the input values and ***g****_j_* is a column vector of length *N* with 1 in the *j*th row, 0 elsewhere.

Covariances and uncertainties for the interpolated values are then found by recognizing that the values of *y_m_* and *y_n_* in Eq. (29) are represented by

$$\begin{array}{l} y_{m}=f_{m}{\left(y_{i}\;y_{i+1}\;y_{i}^{\prime \prime }\;y_{i+1}^{\prime \prime }\;y_{j}\;y_{j+1}\;y_{j}^{\prime \prime }\;y_{j+1}^{\prime \prime }\right)}^{T}\\ y_{n}=f_{n}{\left(y_{i}\;y_{i+1}\;y_{i}^{\prime \prime }\;y_{i+1}^{\prime \prime }\;y_{j}\;y_{j+1}\;y_{j}^{\prime \prime }\;y_{j+1}^{\prime \prime }\right)}^{T} \end{array}$$ (42)

where

$$\begin{array}{l} f_{m}=\left(A_{i}\;B_{i}C_{i}\;D_{i}\;0\;0\;0\;0\right)\\ f_{n}=\left(0\;0\;0\;0\;A_{j}\;B_{j}C_{j}\;D_{j}\right) \end{array}$$ (43)

are sensitivity vectors for *y_m_* and *y_n_* against the eight variables in the vectors shown in Eq. (42). Covariances between the interpolated values are then given by

$$u\left(y_{m},y_{n}\right)=f_{m}^{T}Uf_{n},$$ (44)

where

$$U=\left(\begin{array}{llllllll} u^{2}\left(y_{i}\right) & u\left(y_{i+1},y_{i}\right) & u\left(y_{i}^{\prime \prime },y_{i}\right) & u\left(y_{i+1}^{\prime \prime },y_{i}\right) & u\left(y_{j},y_{i}\right) & u\left(y_{j+1},y_{i}\right) & u\left(y_{j}^{\prime \prime },y_{i}\right) & u\left(y_{j+1}^{\prime \prime },y_{i}\right)\\ & u^{2}\left(y_{i+1}\right) & u\left(y_{i}^{\prime \prime },y_{i+1}\right) & u\left(y_{i+1}^{\prime \prime },y_{i+1}\right) & u\left(y_{j},y_{i+1}\right) & u\left(y_{j+1},y_{i+1}\right) & u\left(y_{j}^{\prime \prime },y_{i}+1\right) & u\left(y_{j+1}^{\prime \prime },y_{i+1}\right)\\ & & u^{2}\left(y_{i}^{\prime \prime }\right) & u\left(y_{i+1}^{\prime \prime },y_{i}\right) & u\left(y_{j},y_{i}\right) & u\left(y_{j+1},y_{i}\right) & u\left(y_{j}^{\prime \prime },y_{i}^{\prime \prime }\right) & u\left(y_{j+1}^{\prime \prime },y_{i}\right)\\ & & & u^{2}\left(y_{i+1}^{\prime \prime }\right) & u\left(y_{i},y_{i+1}^{\prime \prime }\right) & u\left(y_{j+1},y_{i+1}^{\prime \prime }\right) & u\left(y_{j}^{\prime \prime },y_{i+1}^{\prime \prime }\right) & u\left(y_{j+1}^{\prime \prime },y_{i+1}^{\prime \prime }\right)\\ & & & & u^{2}\left(y_{j}\right) & u\left(y_{j+1},y_{j}\right) & u\left(y_{j}^{\prime \prime },y_{j}\right) & u\left(y_{j+1}^{\prime \prime },y_{j}\right)\\ & & & & & u^{2}\left(y_{j+1}\right) & u\left(y_{j},y_{j+1}\right) & u\left(y_{j+1}^{\prime \prime },y_{j+1}\right)\\ & & & & & & u^{2}\left(y_{j}^{\prime \prime }\right) & u\left(y_{j+1}^{\prime \prime },y_{j}^{\prime \prime }\right)\\ & & & & & & & u^{2}\left(y_{j+1}^{\prime \prime }\right) \end{array}\right)$$ (45)

with the lower half symmetric about the diagonal. Only half of this matrix is required. The two right quadrants can be separately multiplied on the right by the column vector (*A_j_ B_j_ C_j_ D_j_*)^T^ and the two results combined into a single eight-element column vector for the final multiplication.

### 4.1 Cubic-Spline Interpolation Examples

Consider again the photometer response curve of Fig. 1, to be integrated over the wavelength range from 360 nm to 830 nm (effectively the photometric response to an equal-energy source). The function was interpolated over the same input range (one less value) but shifted by 2.5 nm and the integral recalculated. Similarly, the function was interpolated to 1 nm intervals and the integral recalculated. Table 2 shows the results for these interpolations, where the uncertainty in the integral was calculated based on 1 % uncertainty in the input values, uncorrelated between values. The consequence of ignoring the correlation between the interpolated values is also shown. Correlations between distant points, introduced through the dependence of the second-derivatives, were negligible (largely because the response curve is relatively smooth), but strong correlations were found between near-neighbours.

**Fig. 3:**
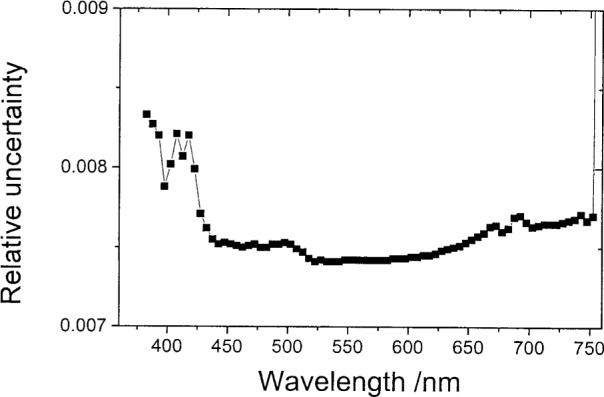
Propagated uncertainty of *V_λ_* for values shifted 2.5 nm; relative uncertainty of the input values is 1 %.

Figure 3 shows propagated uncertainties for the interpolation shifting the input by 2.5 nm; for an interpolation to the mid-point, we would expect the interpolated value for a smooth function to be near the mean of the two interval boundaries with a propagated uncertainty $1/\sqrt{2}=0.71$ of the input (but of course correlated to adjacent values).

**Fig. 4:**
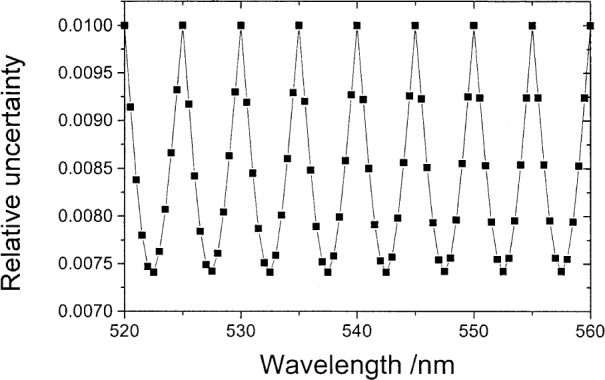
Relative uncertainty of interpolated values for *V_λ_* vs position in the interval. Input values with a relative uncertainty of 1 % are separated by 5 nm.

Figure 4 shows the variation in uncertainty for values interpolated at different positions within the 5 nm interval. This is a practical concern where a wavelength offset may be present in measurements, although in general it is a better practice to retain the wavelength values of the measured points and interpolate weighting functions such as the illuminant or the colorimetric response functions as these carry no uncertainty. At the input points, uncertainties equal that of the input; at the midpoints they fall to $1-\sqrt{2}$ of the input points. Again this is expected as the input data are smooth, and cubic-spline results are not much different from linear interpolation. The cubic-spline reproduces the input ordinate values for abscissa values equal to an input value; for these points, we expect the propagated uncertainty to be that of the input and correlations between similar such values to match that of the input matrix ***U****_y_*. These conditions can be used to test the coding.

**Fig. 5:**
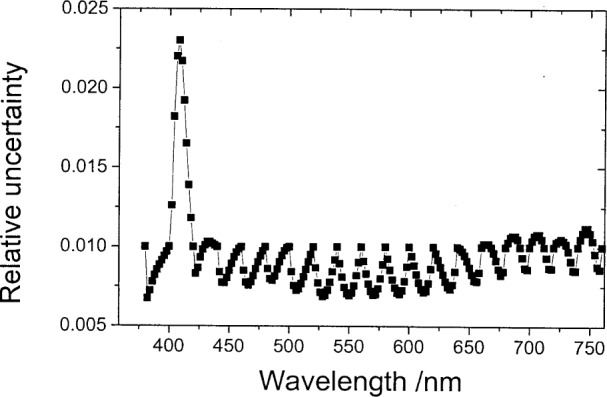
Relative uncertainty for *Vλ* interpolated from a 20 nm grid to 2 nm. Relative uncertainty of the input values set to 1 %.

**Fig. 6:**
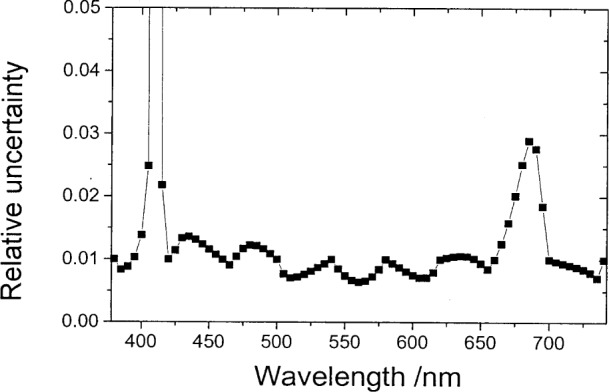
Relative uncertainty for *V_λ_* interpolated from a 40 nm grid to 5 nm. Relative uncertainty of the input values set to 1 %.

**Fig. 7:**
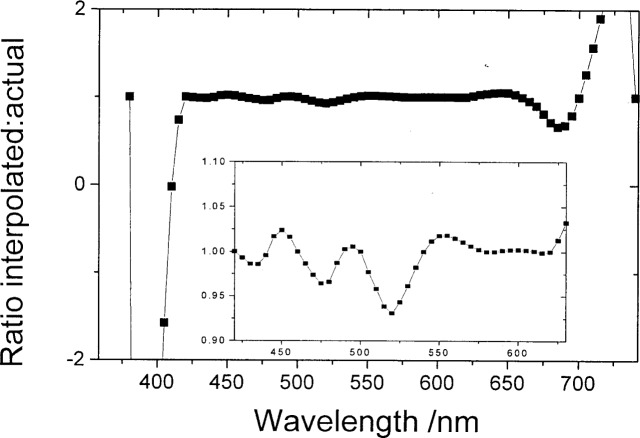
Ratio of *Vλ* interpolated from a 40 nm grid to the actual value on a 5 nm grid. The inset shows the central region on an expanded scale.

Figure 5 shows *V_λ_* interpolated from a 20 nm grid to 2 nm. Where the input curve is changing rapidly relative to the magnitude of the data, linear interpolation would be discontinuous and for these regions, the relative uncertainty of the interpolated values rises above that of the 1 % assumed for the input values. This is shown more strongly in Fig. 6 for input data spaced at 40 nm, interpolated to 5 nm. Here the interpolation does not provide a good representation of *V_λ_* (as shown in Fig. 7); where the interpolation is poor, the uncertainties for the interpolated values rise above those of the input.

## 5. Conclusion

Interpolation of spectral data is a common occurrence in radiometric and photometric measurements. Those data are often then combined in forming integral values such as photometric or colorimetric responses or filter radiometer responses. Interpolation is particularly important when a relatively smooth curve available only on a wide spectral spacing may need to be convolved with a more-rapidly changing curve and then integrated. Uncertainties in the interpolated values will generally be smaller than those of the input data, although this is not always true in the case of cubic-spline interpolation. Uncertainties in combinations calculated using the interpolated data set then must include correlations introduced by the interpolation. Ignoring the correlation will lead to significant underestimation of uncertainties. The calculations in this paper for both Lagrange interpolation and for cubic-spline interpolation show that the uncertainty can be reliably estimated, in practical terms, by propagating the uncertainty through the combination using only the original set of data.

## Figures and Tables

**Table 1:** Integral value and relative uncertainty for various calculation options; four point Lagrange interpolation adding one value in each range. Input values uncorrelated, 1 % relative uncertainty.

Method	Integral	Relative uncertainty
Original data on 5 nm grid	10567.4	0.185
Original data on 10 nm grid	10568.2	0.262
Interpolated data; ignore correlations	10567.5	0.168
Interpolated data; with correlations	10567.5	0.262
Interpolated data; use correlation coefficients	10567.5	0.266

**Table 2:** Integral of V*_λ_* and its uncertainty for input values on a 5 nm grid and 1 % relative uncertainty, uncorrelated

	Integral	Uncertainty	Uncertainty ignoring correlations
Original data	106.8561	0.19647	
Shift 2.5 nm	106.8559	0.19647	0.1459
Interpolate to 1 nm	106.8559	0.19647	0.0746
